# Pemphigus Herpetiformis: A Report of an Unusual Type of Pemphigus in a Three-Year-Old Female

**DOI:** 10.7759/cureus.58286

**Published:** 2024-04-15

**Authors:** Sayed Hashim, Reem Alqusaimi, Reem Rajab, Rawan Almutairi, Humoud Al-Sabah, Atlal Allafi

**Affiliations:** 1 Dermatology, As'ad Al-Hamad Dermatological Center, Kuwait City, KWT

**Keywords:** pemphigus in pediatric, an unusual type of pemphigus, pemphigus, herpetiformis, pemphigus herpetiformis

## Abstract

Pemphigus herpetiformis (PH) is a rare autoimmune blistering disorder that typically presents in adults. However, its occurrence in paediatric patients, especially in very young children, is exceedingly rare. It presents with clinical features resembling dermatitis herpetiformis (DH) and immunologic characteristics similar to pemphigus, belonging to the group of intraepidermal autoimmune bullous diseases. We present the case of a three-year-old female with a history of annular and vesicular lesions on both forearms and legs. A skin biopsy revealed epidermal acanthosis, marked spongiosis, numerous intra-epidermal blisters, and exocytosis of eosinophils and neutrophils. A superficial perivascular lymphocytic infiltrate, accompanied by eosinophils and neutrophils, was also observed in the dermis. The diagnosis was also supported by direct and indirect immunofluorescence. The patient was treated with clobetasol ointment and dapsone, which showed significant improvement in the skin lesions. This case underscores the importance of considering PH in the differential diagnosis of vesicobullous diseases in children and the need for further research to elucidate its pathogenesis and optimal management.

## Introduction

Pemphigus herpetiformis (PH) is a type of vesiculobullous disease that represents 5.62% of all skin lesions in Kuwait [1]. PH is a rare autoimmune blistering disorder that typically presents in adults. However, its occurrence in paediatric patients is exceedingly rare. This condition has clinical aspects comparable to dermatitis herpetiformis (DH) and immunologic characteristics similar to pemphigus. The clinical presentation can vary and may include coalescent annular or gyrate vesiculopustular lesions belonging to the group of intraepidermal autoimmune bullous diseases [2,3]. Herein, we share the case of a three-year-old female who presented with a history of annular and vesicular lesions on both her forearms and legs. A skin biopsy revealed epidermal acanthosis, marked spongiosis, numerous intra-epidermal blisters, and exocytosis of eosinophils and neutrophils.

## Case presentation

**Figure 1:**
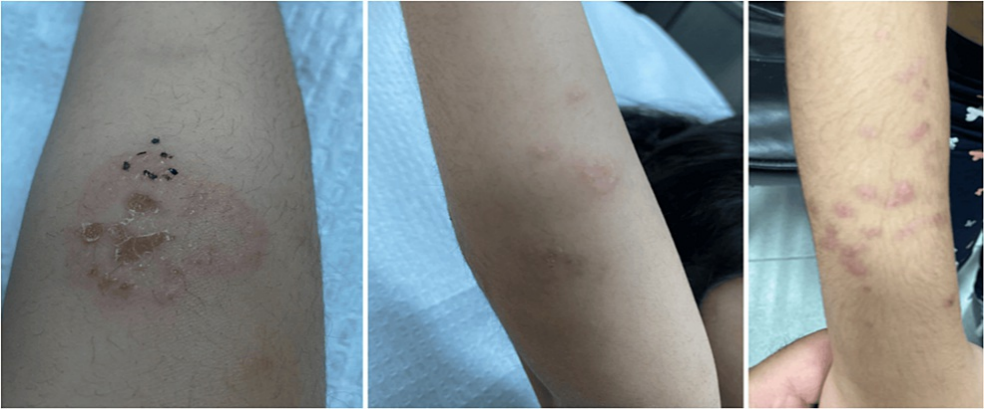
Clinical findings of pemphigus herpetiformis Physical examination showed annular erythematous plaques and vesicles involving both forearms.

A previously healthy, three-year-old Kuwaiti girl with no familial history of autoimmune bullous diseases presented with pruritic annular vesicular lesions that started to appear four months earlier. Physical examination showed annular erythematous plaques and vesicles involving both forearms and legs (Figure 1). Nikolsky's sign was negative. There was neither nail nor mucosal involvement. The physical examination was otherwise unremarkable.

**Figure 2:**
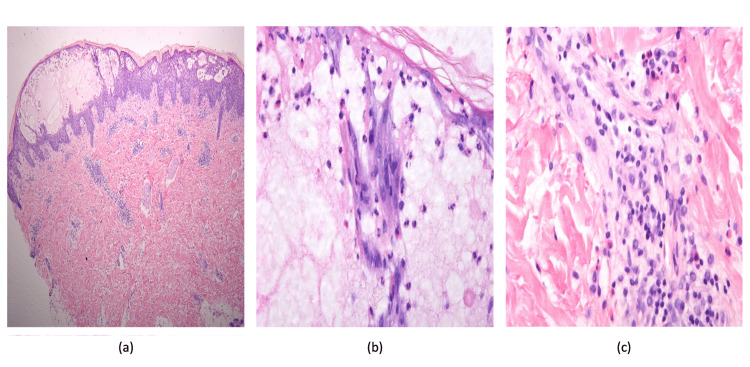
Histopathological examination of skin biopsy using hematoxylin and eosin stain Histopathological evaluation of the skin biopsy revealed epidermal acanthosis, marked spongiosis, numerous intra-epidermal blisters (HE ×10) (a), blister cavity containing eosinophils and neutrophils (HE ×20) (b). Superficial perivascular lymphocytic infiltrate, accompanied by eosinophils and neutrophils, was also observed in the dermis (HE ×40) (c).

Histopathological analysis of a forearm skin biopsy revealed epidermal acanthosis, marked spongiosis, and numerous intra-epidermal blisters. The blister cavity contained eosinophils and neutrophils. A superficial perivascular lymphocytic infiltrate, accompanied by eosinophils and neutrophils, was also observed in the dermis (Figure 2).

**Figure 3:**
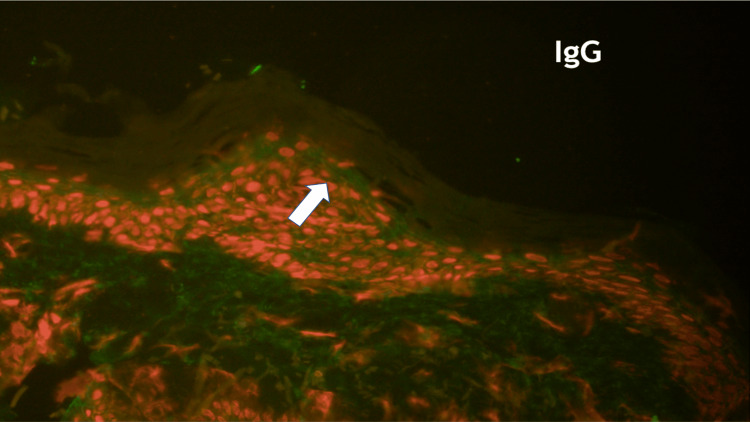
Immunofluorescence examination Through direct immunofluorescence, intercellular IgG deposits were observed within the epidermis of the lesional skin. Arrow indicate the deposition of the IgG antibody.

Through direct immunofluorescence, intercellular IgG deposits were observed within the epidermis of the lesional skin. The substrate for indirect immunofluorescence, the monkey oesophagus, demonstrated intercellular binding of IgG antibodies (Figure 3). Enzyme-linked immunosorbent assays for desmoglein 1 and desmoglein 3 were negative. In accordance with the clinical herpetiform pattern, histological and immunological characteristics were also indicative of PH.

**Figure 4:**
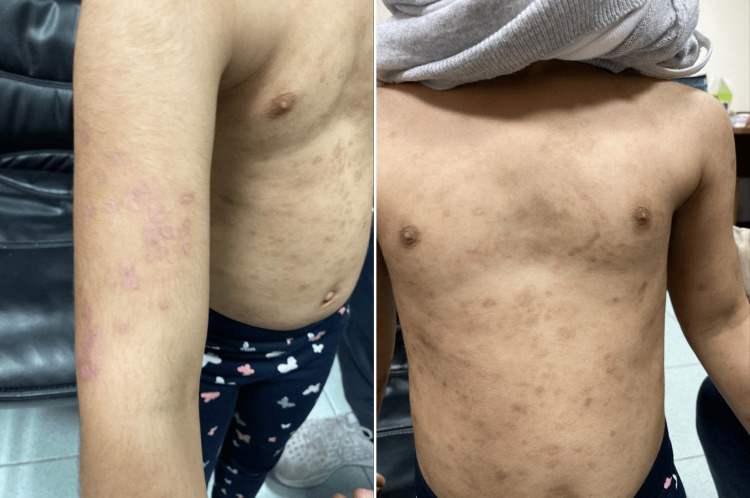
Follow-up after two weeks After two weeks of follow-up, the patient showed little improvement.

**Figure 5:**
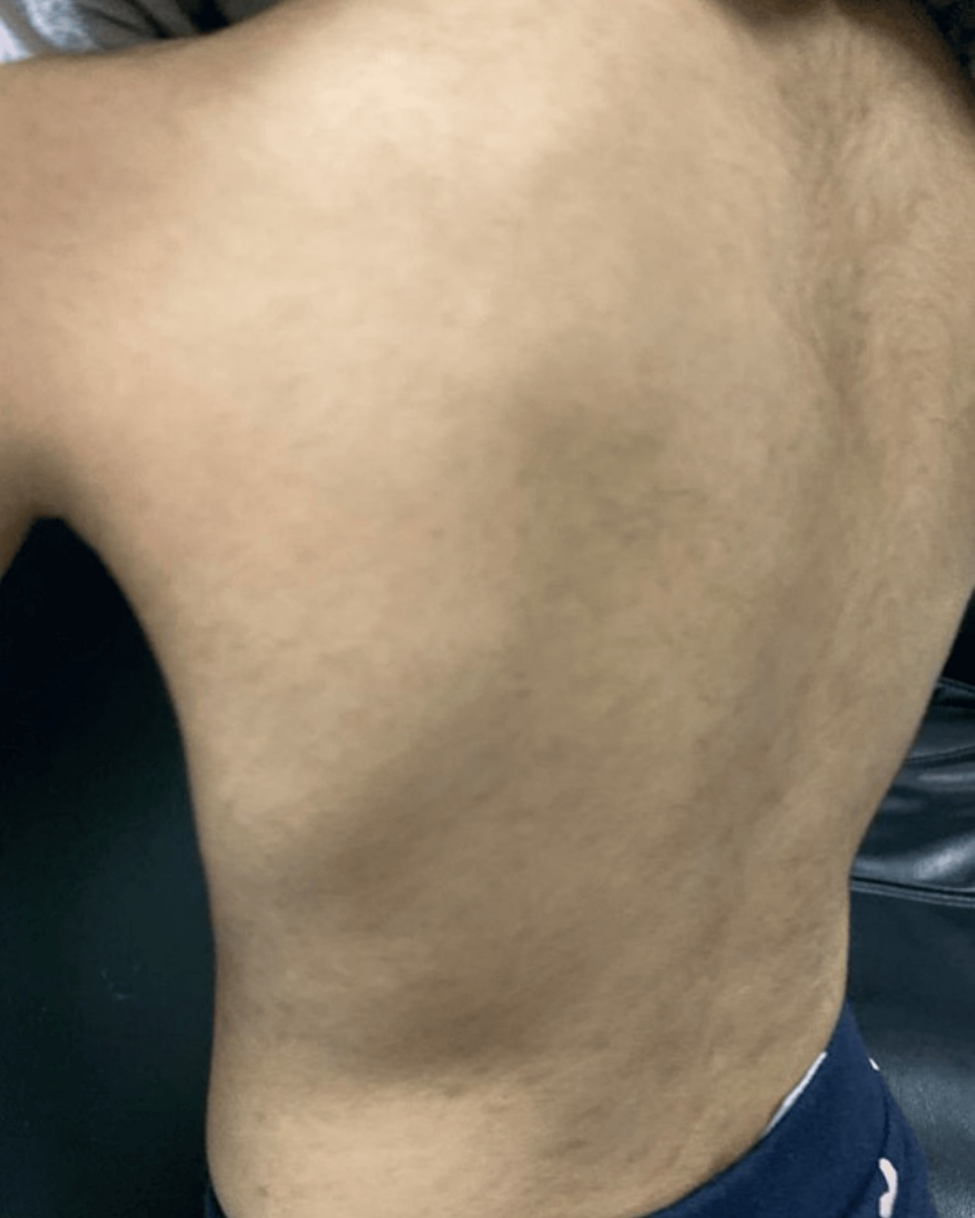
Follow-up after one and half month There was a marked improvement in her skin lesions and her parents were satisfied with the results.

The treatment plan included using Clobetasol ointment once daily for symptomatic relief and dapsone 1 mg/kg daily, after confirming a normal glucose-6-phosphate dehydrogenase (G6PD) level. After two weeks of follow-up (Figure 4), the patient showed little improvement. However, after one and a half months (Figure 5) her skin lesions markedly improved, and her parents were satisfied with the results.

## Discussion

Lesions seen in patients with PH are usually similar to other vesicobullous diseases such as DH, including erythematous, vesicular, bullous, or papular annular-shaped lesions, which usually present in a grouped pattern and are severely pruritic. These lesions commonly affect the trunk and proximal extremities, sparing mucosal membranes in most cases. Other less-involved sites include the neck, scalp, and face. PH initially presents as an atypical eruption with various manifestations, and the diagnoses considered are typically DH, bullous pemphigoid, linear immunoglobulin A dermatosis, pemphigus foliaceous (PF), drug-induced rashes, or allergic contact dermatitis [4-6]. The similarity in clinical presentation necessitates a skin biopsy for histopathological examination and an immunological test to confirm the diagnosis of PH.

Histopathological examination of PH lesions is important to differentiate PH from mimickers. Histopathological characteristics of PH are distinct from those of pemphigus vulgaris and foliaceous pemphigus due to the predominance of spongiosis in the absence of acantholysis. Histological observations may exhibit variability among patients, with a single patient presenting diverse histological characteristics simultaneously. The histology of PH also differs according to the progression of topical lesions. The early to late stages of the disease are distinguished by the presence of eosinophilic spongiosis and subcorneal or intraepidermal neutrophilic and eosinophilic microabscesses. Inflammatory cell infiltration is also present and is composed primarily of eosinophils, neutrophils, or both. Acantholysis, in contrast to pemphigus vulgaris, is a rare observation that may manifest during the advanced phases of the illness [2,6].

Using perilesional skin samples, direct immunofluorescence reveals IgG and C3 deposition surrounding the keratinocyte cell surfaces in the upper epidermis. Indirect immunofluorescence detects the presence of IgG antibodies that bind to the surface of epidermal cells in circulation. Antibodies specifically target desmoglein 1, a protein mostly situated in the upper epidermis, and, to a lesser extent, desmoglein 3, which is found in the lower epidermis [6].

The management of PH requires a comprehensive approach that takes into account the unique characteristics of this rare form of pemphigus. Treatment strategies for PH typically involve a combination of therapies tailored to each patient. Systemic corticosteroids, such as prednisolone, are commonly the first-line treatment for managing bullous pemphigoid and pemphigus diseases. Additionally, medications like dapsone, azathioprine, methotrexate, cyclophosphamide, and hydroxychloroquine may be used as steroid-sparing agents to minimize long-term corticosteroid use [7]. Dapsone, specifically, has been recognized as an effective therapeutic choice for treating DH, a condition that shares similarities with PH [8].

## Conclusions

PH is a rare type of autoimmune blistering disease. Histopathological and immunological examinations are crucial to confirm the diagnosis of PH. Patients with PH respond well to dapsone. This case underscores the importance of considering PH in the differential diagnosis of vesicobullous diseases in children and the need for further research to elucidate its pathogenesis and optimal management in the paediatric population.
